# Genotypic, Developmental and Environmental Effects on the Rapidity of *g_s_* in Wheat: Impacts on Carbon Gain and Water-Use Efficiency

**DOI:** 10.3389/fpls.2019.00492

**Published:** 2019-04-17

**Authors:** Michele Faralli, James Cockram, Eric Ober, Shellie Wall, Alexander Galle, Jeroen Van Rie, Christine Raines, Tracy Lawson

**Affiliations:** ^1^School of Biological Sciences, University of Essex, Colchester, United Kingdom; ^2^The John Bingham Laboratory, NIAB, Cambridge, United Kingdom; ^3^BASF Agricultural Solutions Belgium NV, Ghent, Belgium

**Keywords:** stomatal rapidity, *Triticum aestivum* L., photosynthesis, stomatal conductance, water-use efficiency, water stress, elevated [CO_2_]

## Abstract

Stomata are the primary gatekeepers for CO_2_ uptake for photosynthesis and water loss via transpiration and therefore play a central role in crop performance. Although stomatal conductance (*g_s_*) and assimilation rate (*A*) are often highly correlated, studies have demonstrated an uncoupling between *A* and *g_s_* that can result in sub-optimal physiological processes in dynamic light environments. Wheat (*Triticum aestivum* L.) is exposed to changes in irradiance due to leaf self-shading, moving clouds and shifting sun angle to which both *A* and *g_s_* respond. However, stomatal responses are generally an order of magnitude slower than photosynthetic responses, leading to non-synchronized *A* and *g_s_* responses that impact CO_2_ uptake and water use efficiency (*_i_WUE*). Here we phenotyped a panel of eight wheat cultivars (estimated to capture 80% of the single nucleotide polymorphism variation in North–West European bread wheat) for differences in the speed of stomatal responses (to changes in light intensity) and photosynthetic performance at different stages of development. The impact of water stress and elevated [CO_2_] on stomatal kinetics was also examined in a selected cultivar. Significant genotypic variation was reported for the time constant for stomatal opening (*K_i_, P* = 0.038) and the time to reach 95% steady state *A* (*P* = 0.045). Slow *g_s_* opening responses limited *A* by ∼10% and slow closure reduced *_i_WUE*, with these impacts found to be greatest in cultivars Soissons, Alchemy and Xi19. A decrease in stomatal rapidity (and thus an increase in the limitation of photosynthesis) (*P* < 0.001) was found during the post-anthesis stage compared to the early booting stage. Reduced water availability triggered stomatal closure and asymmetric stomatal opening and closing responses, while elevated atmospheric [CO_2_] conditions reduced the time for stomatal opening during a low to high light transition, thus suggesting a major environmental effect on dynamic stomatal kinetics. We discuss these findings in terms of exploiting various traits to develop ideotypes for specific environments, and suggest that intraspecific variation in the rapidity of stomatal responses could provide a potential unexploited breeding target to optimize the physiological responses of wheat to dynamic field conditions.

## Introduction

Wheat (*Triticum aestivum* L.) is one of the most important food crops globally, accounting for 20% of human calorie consumption (Ray et al., 2013). Significant yield gains have been achieved in the last century following both genetic improvements and advances in crop management (Slafer et al., 2015). However, more recently, evidence of stagnation in yield improvement, combined with the predicted environmental changes associated with global warming (Ray et al., 2012), highlight the need to identify optimized crop ideotypes and new genetic targets for incorporation into current wheat breeding programs to maintain and/or improve future productivity.

Crop yield is the product of the cumulative rates of photosynthesis over the growing season and the subsequent capacity of sinks to accept and store these products (Zelitch, 1982). Although previous work suggested that selecting for elevated photosynthetic rate on a leaf area basis does not always produce significant results in terms of yield (Evans, 1996), free-air concentration enrichment experiments (Long et al., 2006) and bioengineering approaches (Driever et al., 2017) have provided promising results, and highlight the possibility of yield gains via elevated rates of photosynthesis. In many crops, while harvest index and light interception capacity are approaching theoretical maximum (∼0.64 and 0.8–0.9 respectively, Long et al., 2006), the efficiency of energy conversion into biomass (i.e., radiation-use efficiency and thus photosynthesis) still has substantial room for improvement (Long et al., 2006). Most of the intraspecific natural variation in photosynthesis for C_3_ plants is mainly due to differences in biochemical capacity including electron transport rates and carboxylation efficiency (Driever et al., 2014; Carmo-Silva et al., 2017). In addition, under natural dynamic conditions photosynthetic process can also be limited by factors such as activation of Calvin cycle enzymes and/or stomatal dynamics (Lawson and Blatt, 2014; Taylor and Long, 2017; Salter et al., 2019).

Stomata control CO_2_ and water vapor exchange between the leaf and the atmosphere, and thus play a unique role in crop productivity and yield (Lawson et al., 2010, 2012). Stomata respond to environmental changes by modifying pore aperture, and both internal and external signals are involved (Lawson and Blatt, 2014). Although external environmental stimuli (e.g., VPD, light, water availability, heat) often occur in combination, stomata generally open in response to high or increasing light intensity, low CO_2_ concentration [CO_2_] and low vapor pressure deficit (VPD), while stomata close in the opposite conditions (Outlaw, 2003; Lawson et al., 2014). In the field, leaf self-shading, cloud cover and sun angle often lead to rapid changes in photosynthetic photon flux density (PPFD), to which photosynthesis rapidly responds while stomatal responses are an order of magnitude slower (Lawson et al., 2010, 2012; Lawson and Blatt, 2014; Slattery et al., 2018). Slow stomatal responses can lead to (i) reduced *A* due to restricted CO_2_ diffusion during a low to high light transition, or (ii) unnecessary water loss during a high to low light transition when stomata lag behind decreases in *A*. Indeed, recent reports suggested that in wheat stomatal limitation of photosynthesis can be up to 10% (McAusland et al., 2016) leading to potential impacts on crop productivity (Lawson and Blatt, 2014; Taylor and Long, 2017; Vialet-Chabrand et al., 2017; Faralli et al., 2019; Vialet-Chabrand and Lawson, 2019). These findings highlight the advantage of selecting genotypes with fast stomatal responses to changes in irradiance, as rapid stomatal opening can increase photosynthetic rate whilst rapid stomatal closure can enhance water use efficiency at the crop level, leading to increased soil moisture conservation and therefore delay the onset of stress during periods of low rainfall (McAusland et al., 2016; Qu et al., 2016).

Although interspecific variation in stomatal responses to changes in light intensity have been previously reported (Vico et al., 2011; McAusland et al., 2016), to our knowledge there are no reports demonstrating intraspecific variation in the rapidity of stomatal responses in wheat. In addition, there are limited reports on the effects of developmental and environmental factors on stomatal rapidity (e.g., Leakey et al., 2002; Gerardin et al., 2018; Haworth et al., 2018). In particular, climate change has been associated with more frequent periods of water stress (Ray et al., 2012) and a significant increase in atmospheric [CO_2_] (Ainsworth and Rogers, 2007), two environmental conditions that strongly affect both *A*, and *g_s_* and therefore crop productivity. Therefore, the main aims of this work were, (i) to assess the extent of natural variation in the speed of stomatal responses in selected wheat cultivars; (ii) to determine the influence of developmental stage (late vegetative, booting, and post-anthesis stages) on such variation; and (iii) to evaluate the impact of reduced water availability and elevated atmospheric [CO_2_] on the rapidity of stomatal responses. A panel of eight winter wheat genotypes, capturing ∼80% of the United Kingdom single nucleotide polymorphism variability (Gardner et al., 2016), was phenotyped at different developmental stages for stomatal rapidity and photosynthetic capacity. In addition, a selected genotype was used to assess the impact reduced water availability and elevated [CO_2_] on stomatal kinetics.

## Materials and Methods

### Plant Material

Eight elite wheat varieties adapted to the United Kingdom were selected: Alchemy, Brompton, Claire, Hereward, Rialto, Robigus, Soissons, and Xi19. These are the founder lines of the ‘NIAB Elite MAGIC’ multi-parent advanced generation inter-cross (MAGIC) population (Mackay et al., 2014). Seeds were sown in plastic trays containing compost and germinated in a growth cabinet (Reftech BV, Sassenheim, Netherlands) at ∼200 μmol m^-2^ s^-1^ PPFD, 14 h/10 h photoperiod (light/dark), ∼15°C on average and ∼60% relative humidity (RH). The compost material (Levington F2S) contained fertilizer (144 mg L^-1^ N, 73 mg L^-1^ P, 239 mg L^-1^ K, adjusted to pH 5.3–6.0 with dolomitic lime) and incorporated coir and sand. Plants were watered every 2 days. At BBCH (Biologische Bundesanstalt, Bundessortenamt und Chemische Industrie) growth stage (GS) 12 (GS12, two seedling leaves unfolded; Lancashire et al., 1991) seedlings were moved into a cold room for vernalization: 4°C, ∼50 μmol m^-2^ s^-1^ PPFD at 10 h/14 h photoperiod (light/dark) for 8 weeks. After vernalization, seedlings (one per pot) were transplanted into 1.5 L (15 cm diameter; 12 cm deep) or 4 L (16.5 cm diameter; 21 cm deep) pots (depending on the experiment) containing Levington F2S compost (Everris, Ipswich, United Kingdom). After which plants where transferred to either the glasshouse or controlled growth environment depending on experimental design (see below).

### Growth Conditions and Experimental Design

#### Experiment 1: Phenotyping Stomatal Rapidity at Different Developmental Stages

To assess the presence of natural variation for stomatal rapidity and to determine the influence of developmental stage on this trait, plants were grown in a greenhouse in a fully randomized block design, in six blocks (*n* = 6). Solar radiation was supplemented with sodium vapor lamps (∼200 to 400 μmol m^-2^ s^-1^. Hortilux Schreder 600W, Monster, Netherlands) and maintaining a 12 h photoperiod. Air temperature was on average ∼20°C during the day and ∼15°C at night. Water was applied daily to avoid soil moisture deficit, while full strength Hoagland’s nutrients solution (∼100 mL per pot) was applied weekly. Owing to the different developmental pattern of the lines studied in this work, plants were visually scored for growth stage every 2 days. All phenotypic measurements were collected at BBCH GS25-31 (vegetative growth, tillering to start of stem extension), GS41-45 (early reproductive growth, booting stage) and GS71-75 (post-anthesis; ‘watery ripe’ to ‘medium milk’ stages of grain).

#### Experiment 2: Stomatal Rapidity Under Reduced Water Availability

To evaluate the impact of reduced water availability on stomatal dynamics, plants (cv. Soissons) were transplanted into 4 L pots and watered daily to avoid soil moisture deficit until the start of the treatment, and nutrients were supplied with Hoagland’s solution (∼100 mL per pot, until the start of water availability manipulation). Between GS45 and GS51, pots were watered daily to ensure full soil water capacity by weighing the pots (∼3000 g of pot target weight). The non-stressed plants (well-watered, WW, *n* = 6) were watered daily throughout the experiment, whereas the progressive soil drying treatment was applied by removing watering to the water stressed plants (WS, *n* = 6). Water content in the pot was expressed as the fraction of transpirable soil water (FTSW). The FTSW method was recently summarized by King and Purcell (2017), and briefly described as follows: FTSW = (Pg – Pd)/TTSW, where (i) total transpirable soil water (TTSW) was the difference between the pot weights at 100% water holding capacity (WHC) (pot weight ∼3000 g including plant and plastic pot) and when transpiration rate of the stressed plants decreased to 10% of the control plants, (ii) Pg was the actual pot weight on a given date, and (iii) Pd was the pot weight at the time when transpiration rate of stressed plants was 10% of the control plants (∼1300 g of pot weight). Gas exchange analyses were carried out when FTSW was ∼0.2–0.3 for WS plants, and ∼0.8–0.9 for the WW treatment. The value at which WS plants were analyzed was chosen to represent a soil water stress condition at which wheat has previously been found to show typical stress symptoms (e.g., significant reduction of *g_s_*, leaf water potential and leaf relative water content) (Weldearegay et al., 2016). Two sets of soil drying treatments where carried out separately (*n* = 3 for WS for each cycle) to avoid overlaps between replicates during the phenotypic analysis (Supplementary Figure 1).

#### Experiment 3: Stomatal Rapidity Under Elevated Atmospheric [CO_2_]

To evaluate the impact of elevated atmospheric [CO_2_] on the rapidity of stomatal responses a third experiment was carried out in growth chambers in which atmospheric [CO_2_] was manipulated (Conviron Adaptis A1000, Conviron, Canada). Plants (cv. Soissons) were transplanted into in 1.5 L pots (one per pot) and placed into two growth chambers, one set of pots (*n* = 6) at ambient [CO_2_] ([CO_2_] 446 ± 31 μmol mol^-1^ on average) and the other (*n* = 6) at elevated [CO_2_] (706 ± 6 μmol mol^-1^ on average) (Supplementary Figure 2). The light level inside both chambers at leaf height was ∼400–800 μmol m^-2^ s^-1^ with a 12 h photoperiod. Air temperature was maintained at ∼20°C through the day and ∼15°C at night, and RH maintained at ∼60%. Plants were watered every 2 days with Hoagland’s solution (∼100 mL per pot). Phenotypic analyses were carried out at GS25-31 (33–42 days after sowing) as described below (Supplementary Figure 2).

### Phenotypic Analysis

#### Analyses of the Rapidity of *g_s_* to Changes in Light Intensity

**FIGURE 1:**
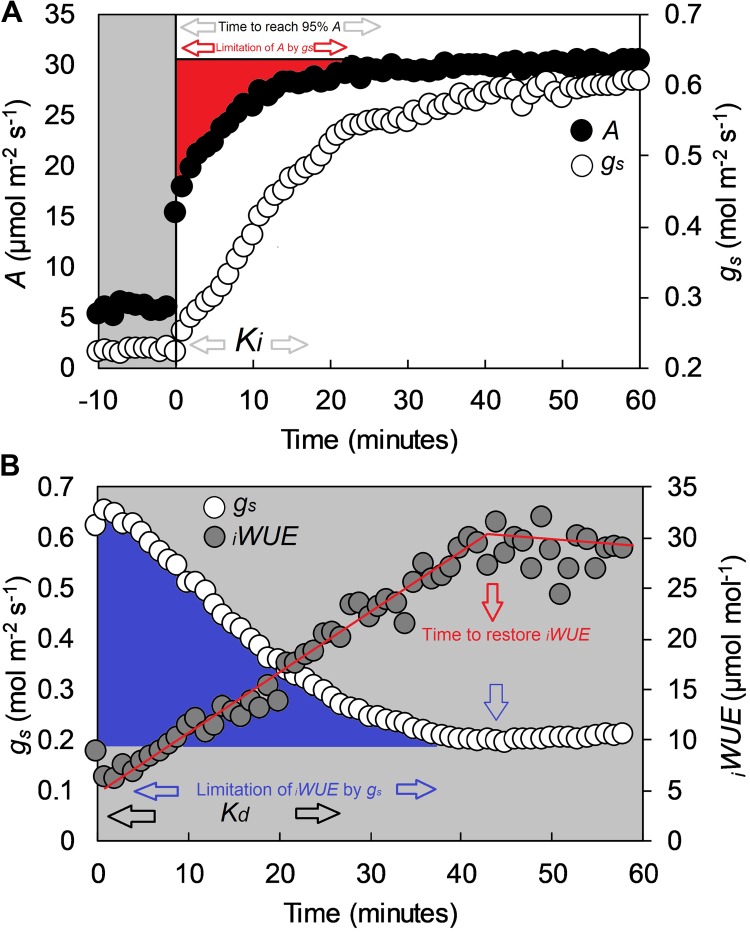
Example of a step-change in light for the flag leaf of a wheat plant (cv. Soissons), collected with a Li-Cor 6400 at GS41. **(A)** Step-change from low to high light (100 to 1500 μmol m^-2^ s^-1^ PPFD), and **(B)** step-change from high to low light (1500 to 100 μmol m^-2^ s^-1^ PPFD). In **(A)**, black dots represent CO_2_ assimilation rate (*A*), whereas white dots represent stomatal conductance (*g_s_*). In **(B)**, white dots represent stomatal conductance (*g_s_*) while gray dots represent intrinsic water-use efficiency (*_i_WUE*) calculated as *_i_WUE* = *A*/*g_s_*. White areas represent 1500 μmol m^-2^ s^-1^ PPFD, gray areas 100 μmol m^-2^ s^-1^ PPFD. Estimated variables with the exponential model described by Vialet-Chabrand et al. (2013) are *K_i_* (time constant for rapidity of stomatal opening), *K_d_* (time constant for rapidity of stomatal closing) and time to reach 95% *A* (T_95%_*_A_*.) The limitation of *A* by *g_s_* (*g_s_*_limit_*A*) was estimated by assuming a hypothetical *A* if no stomatal limitation was present immediately after a low to high light transition (McAusland et al., 2016). Time to restore *_i_WUE* (T*_iWUE_*) was calculated with segmented regression, and estimated as the intercept between the two linear segments. The *g_s_* at the two *_i_WUE* intercepts were used to calculate the limitation of *_i_WUE* by *g_s_* (*g_s_*_limit_*_i_WUE*) by assuming an instantaneous stomatal closure after a high to low light transition.

In each experiment, the third fully expanded leaf at GS31, and the flag leaf at GS41 and GS71 were tagged on each plant at the onset of each selected growth stage. Prior to gas exchange analysis, plants were transferred from the greenhouse to a temperature and humidity-controlled room (∼20°C temperature and ∼60% RH) and gas exchange measurements performed on the middle of the leaf lamina using an open infrared gas exchange system fitted with a 2 cm^2^ leaf cuvette and integral blue–red LED light source (LI-6400–40; LI-COR, Lincoln, NE, United States). All measurements were collected between 8:30 and 15:00 and randomized to avoid any potential diurnal influence over a 8 week measurement period. Prior to measurement, leaves were first equilibrated at a PPFD of 100 μmol m^-2^s^-1^ until both *A* and *g_s_* reached ‘steady state,’ defined as a ∼2% maximum change in rate during a 10 min period (generally 60 min). After equilibration, PPFD was increased to 1500 μmol m^-2^s^-1^ for 1 h, and subsequently returned to 100 μmol m^-2^s^-1^ for 1 h. The conditions inside the leaf cuvette were kept constant at 20 ± 0.1°C leaf temperature, at VPD of 1 kPa with a dew point generator (LI-610; LI-COR, Lincoln, NE, United States) and at 400 μmol CO_2_ mol^-1^ air (ambient CO_2_ concentration, *C_a_*). In Experiment 3 the plants grown at ∼700 μmol mol^-1^ [CO_2_] were analyzed at 700 μmol mol^-1^ *C_a_.* Values were logged every minute throughout the three h measurement cycle. Intrinsic water use efficiency (*_i_WUE*) was calculated as *_i_WUE* = *A*/*g_s_*. All data were analyzed according to the exponential model of Vialet-Chabrand et al. (2013) as described in McAusland et al. (2016). Variables estimated with the exponential model were steady-state photosynthesis at saturating light (*A*), steady state stomatal conductance at saturating light (*g_s_*), *K_i_* (time constant for rapidity of stomatal opening), *K_d_* (time constant for rapidity of stomatal closing) and ‘time to reach 95% *A*’ (T_95%_*_A_*) (Figure 1A,B). The limitation of *A* by *g_s_* (*g_s_*_limit_*A*) was calculated by estimating a hypothetical *A* if no stomatal limitation was present (McAusland et al., 2016) and determining the differences with the measured kinetic values. The ‘time to restore *_i_WUE*’ (T*_iWUE_*) was defined as the time necessary to recover the maximum *_i_WUE* value during the high to low light transition. T*_iWUE_* was calculated using segmented regression and estimated as the intersection between the two linear segments (Figure 1B). The *g_s_* at the point of intercept was used to determine the ‘limitation of *_i_WUE* by *g_s_*’ (*g_s_*_limit_*_i_WUE*) by calculating the integrated difference with measured values following the high to low light transition (Figure 1B).

#### A/Ci Curves

Photosynthesis measurements (*A*/*C_i_* curves) were performed between 9:00 and 12:00 on the fully emerged flag leaf at GS41-45 in Experiment 1. Measurements of the response of *A* to sub-stomatal CO_2_ concentrations (*C_i_*) were performed in the middle of the tagged leaf using an open infrared gas exchange system and a 2 cm^2^ leaf cuvette with an integral blue–red LED light source (LI-6400–40; LI-COR, Lincoln, NE, United States). In the cuvette, PPFD was maintained at a saturating level of 1500 μmol m^-2^s^-1^, a leaf temperature of 20 ± 0.1°C, a VPD between 0.9 and 1.3 kPa and a *C_a_* of 400 μmol mol^-1^. When steady-state conditions were achieved, *C_a_* was sequentially decreased to 300, 200, 100, and 75 μmol mol^-1^ before returning to the initial concentration of 400 μmol mol^-1^. This was followed by a sequential increase to 550, 700, 1000, and 1200 μmol mol^-1^. Readings were recorded when *A* had stabilized to the new conditions. The maximum velocity of Rubisco for carboxylation (*V_cmax_*) and the maximum rate of electron transport demand for Ribulose 1,5-bisphosphate (RuBP) regeneration (*J_max_*) were derived by curve fitting, as described by Sharkey et al. (2007).

#### Stomatal Density Analysis

At GS41-45 in Experiment 1, stomatal impressions were collected at the same point of the leaf lamina used for gas exchange analyses, on both the adaxial (*n* = 6) and abaxial (*n* = 6) side of the leaf. A negative impression was made using a dental polymer (Xantoprene, Heraesus Kulzer, Ltd., Hanau, Germany) (Weyers and Johansen, 1985). After the material had dried, a positive impression was produced using nail polish on a microscope slide. Stomatal density and pore length were determined using a light microscope by averaging the value of six fields of view for each leaf with a size of ∼1250 μm^2^ captured from each impression and using a 5 MP eye-piece camera (MicroCAM 5 MP, Bresser Optics, Rhede, Germany).

### Statistical Analysis

Statistical analyses were conducted using SPSS (v.16; SPSS, Inc., Chicago, IL, United States) and R^1^. A two-way analysis of variance (ANOVA) was used for gas exchange data when two factors (genotype × growth stage) were present (i.e., for the variables *A, g_s_, K_i_, K_d_*, T_95%_*_A_, g_s_*_limit_*_A_*, T*_iWUE_* for Experiment 1). Single factor analyses were carried out using one-way ANOVA (i.e., for *A, g_s_, K_i_, K_d_* in Experiments 2 and 3). Shapiro–Wilk and Levene’s tests were used to test data for normality and homogeneity of variance, respectively. Duncan’s test was used for multiple comparisons. When present, linear curves were fitted with major axis regression thus minimizing the variability for the traits of interest in both the axes. The strength of trait associations at GS41-45 (between steady-state and dynamic gas exchange, anatomical and photosynthetic capacity traits) and for all developmental stages (between steady-state and dynamic gas exchange traits) were measured using Pearson’s correlation coefficient.

## Results

### Speed of Stomatal Responses at Different Developmental Stages

**FIGURE 2:**
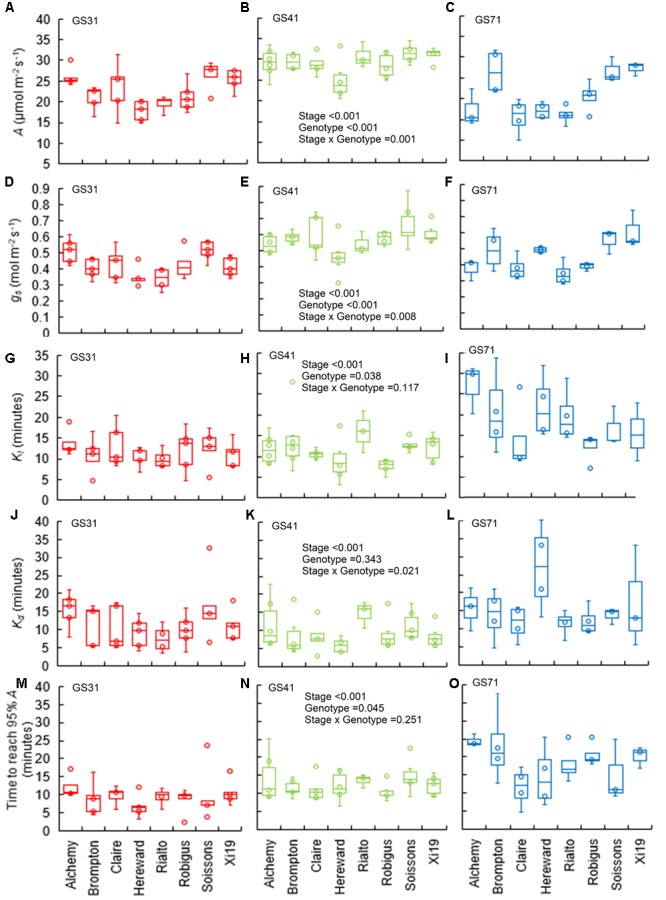
Box plots for steady-state and estimated parameters from step-changes in light at three growth stages on a panel of eight wheat genotypes. Data were collected at collected at GS31, GS41, and GS71 respectively (see graph); **(A–C)** *A*, CO_2_ assimilation rate at saturating light after 60 min of induction at 1500 μmol m^-2^ s^-1^ PPFD. **(D–F)** *g_s_*, stomatal conductance at saturating light after 60 min of induction at 1500 μmol m^-2^ s^-1^ PPFD. **(G–I)** *K_i_*, time constant for stomatal opening. **(J–L)** *K_d_*, time constant for stomatal closure. **(M–O)** ‘Time to reach 95% *A*’ (T_95%_*_A_*). Data were analyzed using two-way ANOVA and means separation was carried out with Duncan’s test (Supplementary Table S1). All data are means of *n* = 4–7.

Significant genotypic variation (*P* < 0.001) in steady-state *A* and *g_s_* at 1500 μmol m^-2^ s^-1^ was recorded for the eight wheat cultivars investigated (Figure 2A–F). Soissons and Xi19 showed the highest *A* and *g_s_* values, whereas Hereward showed consistently lower values. There was a significant effect of growth stage on *A* and *g_s_* (*P* < 0.001 for both), with most of the cultivars showing higher values at GS41. Significant variation in the time constants for stomatal opening (*K_i_*) was recorded between cultivars (*P* = 0.038) (Figure 2G–I) and developmental stage significant impact *K_i_* (*P* < 0.001) in the majority of cultivars, with a lower time constant (thus faster *g_s_* responses) at GS31 and GS41 compared with GS71. However, in cultivars Claire, Rialto and Robigus, there was no significant effect of growth stage on *K_i_*. Similarly, *K_d_* varied significantly between the different growth stages (*P* < 0.001), although no significant genotypic differences were found (*P* = 0.343) (Figure 2J–L). Most of the cultivars achieved 95% *A* between 7 and 15 min following a step increase in light intensity when analyzed at GS31 and GS41 and significant variation (*P* = 0.045) existed between cultivars (Figure 2M–O). At GS71, T_95%_*_A_* was significantly longer than GS31 and GS41 (*P* < 0.001), between 14 and 25 min.

**FIGURE 3:**
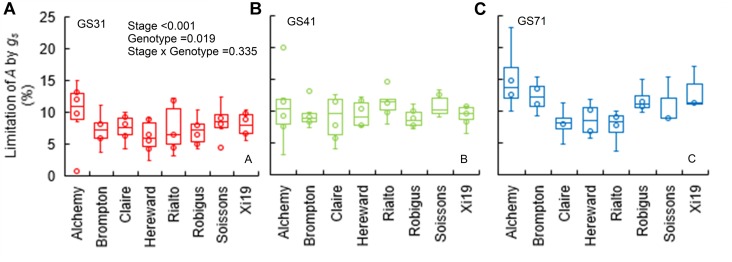
Limitation of *A* by *g_s_* (*g_s_*_limit_*A*) after 30 min of the step change from low to high light (100 to 1500 μmol m^-2^ s^-1^ PPFD) assessed for eight wheat genotypes over three key stages of development (GS31, GS41, and GS71 as **A–C** respectively). Data were estimated by assuming a hypothetical *A* if no stomatal limitation was present immediately after a low to high light transition. Data were analyzed using two-way ANOVA [means separation was carried out with Duncan’s test (Supplementary Table S1)]. Data are means (*n* = 4–7).

When plants were subjected to a step increase in light intensity, photosynthesis was limited by the slow increase in *g_s_*, with an average limitation (*g_s_*_limit_*A*) between 7 and 15% across genotypes (*P* = 0.019) and growth stages (Figure 3A–C). Soissons and Alchemy showed the greatest limitation of *A* by *g_s_* (∼12% on average) while, Rialto, Hereward and Claire were less limited at ∼8% on average. Generally, *g_s_*_limit_*A* was exacerbated at GS71 (*P* < 0.001), although some genotypes (Claire, Rialto) did not show any significant increases in *g_s_*_limit_*A* at GS71 compared to GS41.

**FIGURE 4:**
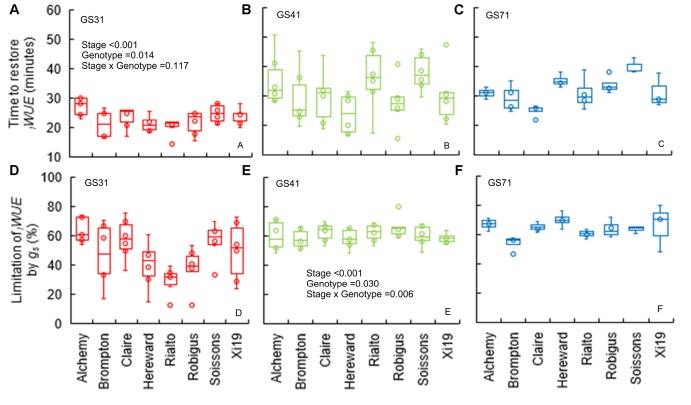
Time to restore *_i_WUE* (T*_iWUE_*) **(A–C)** and limitation of *_i_WUE* by *g_s_* (*g_s_*_limit_*_i_WUE*) **(D–F)** of the step change from high to low light (1500 to 100 μmol m^-2^ s^-1^ PPFD) assessed for eight wheat genotypes over three key stage of development (GS31, GS41, and GS71). Time to restore *_i_WUE* was calculated with segmented regression, and estimated as the intercept between the two linear segments. The *g_s_* at the two *_i_WUE* intercepts was used to calculate the limitation of *_i_WUE* by *g_s_* by assuming an instantaneous stomatal closure after a high to low light transition. Data were analyzed by using two-way ANOVA [means separation was carried out with Duncan’s test (Supplementary Table 1)] and shown as means (*n* = 4–7).

**FIGURE 5:**
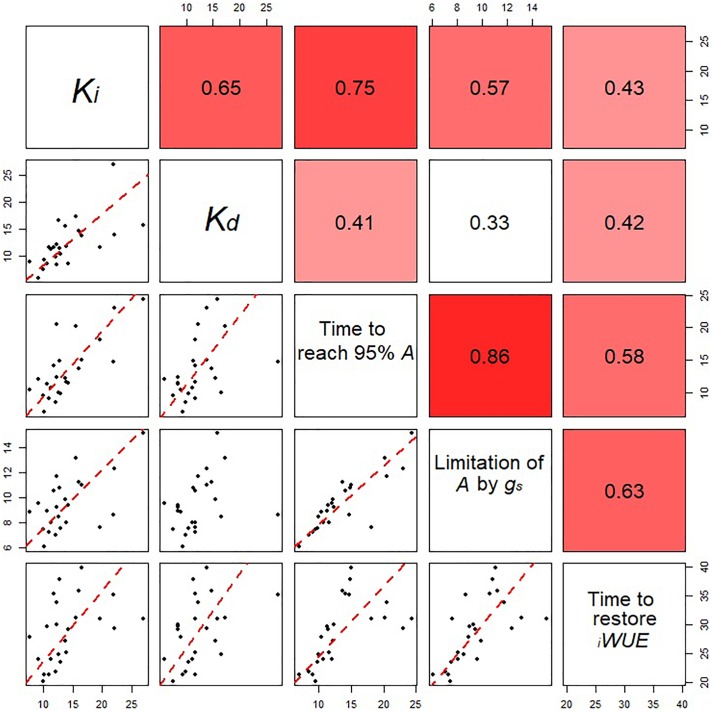
Relationships between the estimated parameters for stomatal opening and closing for all eight wheat varieties at all the growth stages analyzed. Data points are means (*n* = 4–7) for the eight cultivars at three different growth stages. Correlation coefficients between parameters are shown in the top right panels. In the bottom panels, regression was fitted using major axis regression. Fitting lines are shown only when the correlation is significant (*P* < 0.05).

The time to restore *_i_WUE* (T*_iWUE_*) was generally faster at GS31 compared to GS41 and GS71 (*P* < 0.001) (Figure 4A–C). Hereward was the quickest to restore *_i_WUE* due to fast stomatal closure (low *K_d_*) at GS31 and GS41, whilst the slowest responses were observed in Alchemy at GS31 and Soissons at both GS41 and GS71 (*P* = 0.014). *g_s_*_limit_*_i_WUE* was significantly different between cultivars (*P* = 0.030) and growth stages (*P* < 0.001) (Figure 4D–F). Across all of the growth stages measured, the temporal response of *g_s_* for opening and closing were significantly correlated with T_95%_*_A_* and *g_s_*_limit_*A* (Figure 5). At the same time, significant correlations were found between T*_iWUE_* and the time constant for stomatal closing.

### Photosynthetic Capacity at Flag Leaf Stage

**FIGURE 6:**
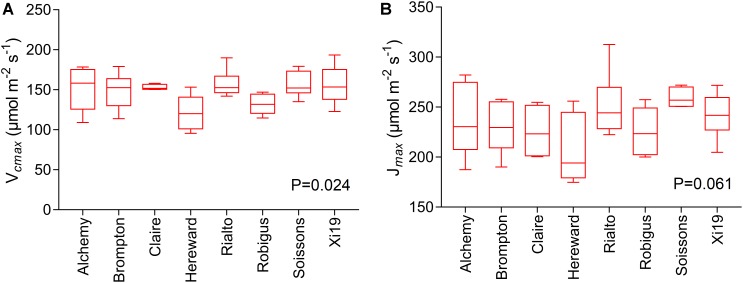
Measures of photosynthetic capacity for the flag leaf of the wheat panel at GS41-45, estimated through *A/C_i_* curves for eight wheat varieties. Data are means (*n* = 5–6 ± standard error of the means). **(A)** The maximum velocity of Rubisco for carboxylation (*V_cmax_*). **(B)** The maximum rate of electron transport demand for RuBP regeneration (*J_max_*).

**FIGURE 7:**
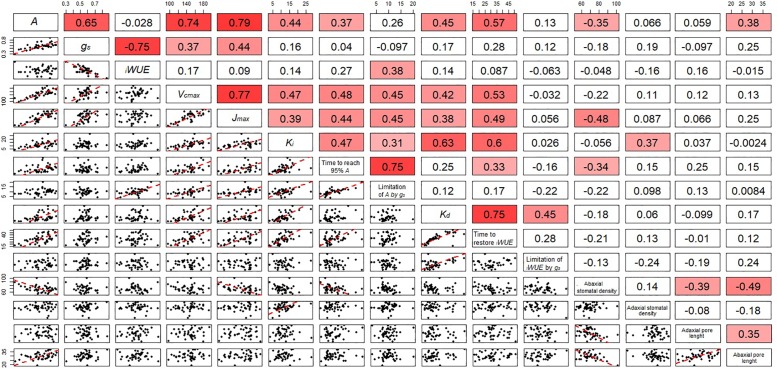
Correlation matrix including the correlation coefficient between parameters describing the temporal response of *g_s_* during opening and closing, photosynthetic capacity and anatomical features for stomata of the flag leaf of wheat plants at GS41. In the bottom panels, regression was fitted by using major axis regression. Fitting line is shown only when the correlation was significant (*P* < 0.05).

Significant genotypic variation in *V_cmax_* (*P* < 0.024) was observed within the eight cultivars analyzed (Figure 6A,B). Rialto, Soissons, and Xi19 showed the highest values for both *V_cmax_* and *J_max_* (∼160 and 260 μmol m^-2^s^-1^ on average, respectively) whereas Robigus and Hereward displayed the lowest values. Significant positive correlations were observed between photosynthetic capacity traits (*A, V_cmax_, J_max_*), *g_s_*, speed of stomatal responses and stomatal density (Figure 7). A significant positive relationships was observed between *g_s_* and *A* whilst a negative relationship between *g_s_* and *_i_WUE* was recorded. In addition, *A* was significantly and positively correlated with most of the stomatal kinetics related traits (*K_i_, K_d_*, T_95%_*_A_, g_s_*_limit_*A*). Interestingly, *_i_WUE* positively correlated with the *g_s_*_limit_*A*. Significant and positive correlations were found between the *g_s_*_limit_*A*, T_95%_*_A,_* and *K_i_*.

### Stomatal Anatomical Features at Flag Leaf Stage

Stomatal density and pore length were significantly different between the cultivars (*P* = 0.002 for abaxial and *P* < 0.001 for adaxial stomatal density while *P* = 0.013 for abaxial and *P* = 0.001 for adaxial pore length) (Table 1). The abaxial density ranged from 63.7 to 81.6 stomata mm^-2^ while the adaxial density was between 61.0 and 90.4 mm^-2^. Stomatal density was correlated with *K_i_* (adaxial, positive) and T_95%_*_A_* (abaxial, negative) (Figure 7) while abaxial pore length was negatively correlated with abaxial stomatal density (Figure 7).

**Table 1:** Stomatal density and pore length for wheat flag leaf analyzed on both the abaxial and the adaxial surface (*n* = 6) and in the eight wheat cultivars.

	Abaxial stomatal density (mm^-2^)	Adaxial stomatal density (mm^-2^)	Abaxial pore length (μm)	Adaxial pore length (μm)
Alchemy	65.6 a	77.9 bc	29.9 bc	31.3 cd
Brompton	81.6 c	90.4 d	25.4 a	26.2 a
Claire	64.2 a	78.1 bc	27.0 ab	28.5 abc
Hereward	76.6 bc	80.3 bcd	24.3 a	27.2 ab
Rialto	65.6 a	73.4 b	26.5 ab	28.5 abc
Robigus	68.7 ab	61.0 a	27.4 abc	30.7 bcd
Soissons	63.7 a	88.3 cd	28.6 abc	32.7 d
Xi19	63.7 a	72.4 b	31.5 c	33.7 d
d.f.	40	40	40	40
*P*-value	0.002	<0.001	=0.013	=0.001


*Data were analyzed by using one-way ANOVA and different letters represent significant differences according to Duncan’s test.*

### Speed of Stomatal Responses Under Reduced Water Availability

**FIGURE 8:**
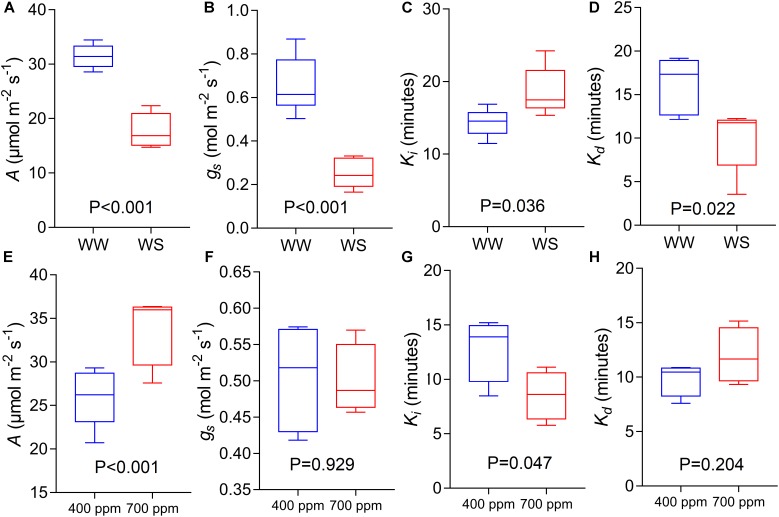
CO_2_ assimilation rate at saturating light after 60 min of induction at 1500 μmol m^-2^ s^-1^ PPFD **(A,E**, *A*), stomatal conductance at saturating light after 60 min of induction at 1500 μmol m^-2^ s^1^ PPFD **(B,F**, *g_s_*), time constant for stomatal opening **(C,G**, *K_i_*), and time constant for stomatal closing **(D,H**, *K_d_*) estimated from step-changes in light collected in Experiments 2 and 3 (well-watered conditions and reduced water availability, WW and WS respectively, **A–D**; ambient and elevated atmospheric [CO_2_] conditions, 400 and 700 μmol mol^-1^ respectively, **E–H**) collected at GS45-51 and GS31 respectively on cv. Soissons. Data were analyzed with one-way ANOVA and shown as means (*n* = 4–6 ± standard error of the means).

Using the variety Soissons, reduced water availability significantly reduced *A* and *g_s_* at 1500 μmol m^-2^s^-1^ PPFD by 45 and 63% respectively (*P* < 0.001) (Figure 8A,B). The time constant *K_i_* was increased (*P* = 0.036) in plants grown under water stress (WS) conditions compared to the well-watered controls (WW) (Figure 8C). In contrast, a significantly lower *K_d_* (*P* = 0.022) was recorded under WS compared with WW (Figure 8D).

### Speed of Stomatal Responses Under Elevated [CO_2_]

The cv. Soissons grown under 700 μmol mol^-1^ [CO_2_] showed a 25% increase in *A* compared to the rate in control plants grown at 400 μmol mol^-1^ [CO_2_] (Figure 8E). In contrast, a small reduction in *g_s_* (6%) was recorded under elevated [CO_2_], although this was not significantly different from *g_s_* at 400 μmol mol^-1^ [CO_2_] (Figure 8F). Elevated [CO_2_] significantly reduced *K_i_* (*P* = 0.047), while no differences were found for *K_d_* (Figure 8G,H).

## Discussion

### Genotypic Variation for Stomatal Rapidity in Wheat

Previous work has demonstrated the presence of significant interspecific (Vico et al., 2011; McAusland et al., 2016) and intraspecific (e.g., rice, Qu et al., 2016) variation in the rapidity of stomatal responses or photosynthetic induction (Salter et al., 2019) in crops. Here, we show that significant genotypic variation in the rapidity of *g_s_* is present in wheat in response to step changes in irradiance. Consistent with the conclusions of previous work (e.g., Vico et al., 2011; Lawson et al., 2012; McAusland et al., 2016), the time to reach maximum steady state *g_s_* ranged from 7 to 27 min between cultivars. Cultivars with faster *g_s_* opening responses (lower *K_i_*) (e.g., Hereward, Claire) achieved 95% *A* more rapidly than those cultivars with slower *g_s_* kinetics (e.g., Xi19, Soissons) supported by the positive correlation between *K_i_* and T_95%_*_A_*. At the same time, cultivars with faster stomatal closing (lower *K_d,_* e.g., Hereward and Claire at GS41) following a high to low light transition achieve a higher *_i_WUE*, more rapidly. These findings support previous reports in which the ‘speedy stomata’ trait has been considered a potential target for maximizing CO_2_ diffusion and *A*, as well as *_i_WUE*, particularly under dynamic light regimes (Lawson and Vialet-Chabrand, 2018). Significant differences in stomatal density and pore length were also observed between cultivars; interestingly, the variation in stomatal density was greater on the adaxial than the abaxial surface. However, only a weak correlation between stomatal density (adaxial) and *K_i_* was apparent, indicating minimal anatomical influence on the speeds of *g_s_* response in the panel of wheat cultivars analyzed. Additionally, our findings are contrary to previous research on the relationship between stomatal speed and density in the non-domesticated species of the dicot genus *Banksia* that have reported higher stomatal density results in faster responses (Drake et al., 2013).

In rice (Qu et al., 2016) and other species (McAusland et al., 2016), asymmetric stomatal responses (e.g., faster stomatal closure that opening) have been suggested as a strategy of prioritizing water conservation over CO_2_ uptake. In our work, the relatively conserved ratio of *K_i_*:*K_d_* (at all growth stages) in all the cultivars studied indicates a balance between carbon gain and water conservation. However, although the time constant for opening and closure were comparable, the fact that T*_iWUE_* was significantly higher than T_95%_*_A_* indicates that slow *g_s_* responses had a greater impact on *_i_WUE* than *A*. While the varieties studied in this work are adapted to a north–west European environment (Mackay et al., 2014), and therefore likely optimized for carbon gain rather than *_i_WUE*, wheat cultivars adapted to lower rainfall regimes may provide a more extensive natural diversity for water conservation (i.e., faster stomatal closure rather than opening). To our knowledge, this is the first report demonstrating natural variation in the speed of stomatal responses in wheat at the leaf level. However, new cutting-edge technologies, for example whole plant gas-exchange (Jauregui et al., 2018), would enable the impact of the speed of stomatal responses on whole plant net carbon assimilation and water use to be evaluated. Cultivars with fast *g_s_* responses (e.g., Claire, Robigus, and Hereward) were followed by lower *A* and *g_s_* values thus showing potential elevated adaptation to dynamic light environment and potentially water deficit conditions. In contrast, Soissons and Xi19 demonstrated high overall *g_s_* values, but slow *g_s_* responses, traits that may be useful for environments in which light is high and constant with higher temperatures but with sufficient water to support high *g_s_* and evaporative cooling. These data suggest that phenotyping wheat lines for stomatal rapidity has the potential to identify novel targets for improving wheat productivity for exploitation in breeding programs.

### Photosynthetic Capacity and Speed of Stomatal Responses

In our study, a significant variation for V*_cmax_* and steady-state *A* and *g_s_* was found between cultivars, consistent with previous studies in wheat (e.g., Driever et al., 2014; Gaju et al., 2016). However, cultivars with greater *g_s_* rapidity displayed lower photosynthetic capacity demonstrated by the positive relationship between *A* and *g_s_* with T_95%_*_A_*, and the time constants for stomatal opening and closing (*K_i_* and *K_d_*), respectively. This suggests a compromise between the rapidity of stomatal behavior and the values of steady state *A* and *g_s_* achieved. Stomatal movement involves a series of hierarchical processes based on the transport, accumulation, and release of osmotically active solutes (Lawson and Blatt, 2014) as well as subsidiary cell physiology (Raissig et al., 2017), and any variation in these processes could result in differences in stomatal behavior. For example, variation in vascular connectivity (e.g., vein density) could explain the positive relationship between steady state *A* and *g_s_* and the speed of *g_s_*. Feldman et al. (2017) recently showed stronger photosynthetic performance in rice with increased leaf vein densities via mutagenesis. It is therefore conceivable that concurrent improvements for stomatal rapidity, photosynthetic capacity and for maximum *A* and *g_s_* could be attained if vein density and hydraulic efficiency were improved.

### Leaf Age Affects the Rapidity of *g_s_* in Wheat

A novel finding of this work is the significant effect of growth stage on stomatal responses. The rapidity of *g_s_* was reduced at post-anthesis stage (GS71) compared to the earlier developmental stages (GS31 and GS41) and this corresponded with a significant decrease in both steady-state *A* and *g_s_*. The decrease in post-anthesis photosynthetic capacity, and therefore reduction in radiation use-efficiency in cereals, has been reported previously (Bingham et al., 2007; Carmo-Silva et al., 2017), and mainly attributed to the onset of leaf senescence (Gaju et al., 2016). The activation of the senescence signaling pathway, thought to be triggered by sink feedback (e.g., Bingham et al., 2007), leads to degradation of chlorophyll and Rubisco and subsequent re-allocation of nutrients from the senescing parts (i.e., leaves) to the growing sink (i.e., grain), thus leading to reduction in the efficiency of the source (Camargo et al., 2016). However, to our knowledge, this is the first report showing developmental effects on stomatal responses to changes in light intensity. In particular, the data highlight growth stage- and genotype-dependent variation in stomatal rapidity, and the importance of taking into account these variables when quantifying dynamic stomatal traits. Additionally, periods of low precipitation and/or high temperature are more common during the post-anthesis stage, often leading to significant yield reductions. Faster stomatal opening could facilitate greater utilization of sudden increases in irradiance, and thus not only provide more assimilates for grain filling, but avoid any potential damage from excess excitation pressure (Yamasaki et al., 2002). Increased *A* is particularly important in view of the potential source-limitation (or at least source-sink co-limitation), which has been reported during grain filling in several wheat cultivars (Álvaro et al., 2008; Acreche and Slafer, 2009). Additionally, as wheat is extremely sensitive to temperature (Yamasaki et al., 2002) rapid *g_s_* responses to increasing irradiance will facilitate maintenance of nearer optimal leaf temperatures to support maximum photosynthetic performance (Lawson and Vialet-Chabrand, 2018).

On the other hand, water conservation strategies would be enhanced by faster stomatal closure when carbon gain is reduced (e.g., during high to low light transition), thus improving the water-use budget and helping to reduce early soil water exhaustion (Bodner et al., 2015). For example, Hereward, Claire, and Robigus showed very quick *g_s_* responses overall with minimal developmental effects (apart from Hereward at GS71), thus being good candidates for breeding exploitation for stomatal rapidity. The fact that a significant variation was observed for *K_i_*, as well as a stage × genotype interaction for *K_d_* and T*_iWUE_*, suggests that the targeted exploitation of existing natural variation could be used to facilitate carbon gain for photosynthesis and optimize water-use under dynamic field environments and at different stages of wheat development.

### Water Stress and Elevated CO_2_ Concentration Affects Stomatal Rapidity

The effect of elevated [CO_2_] and water stress on stomatal rapidity has received little attention to date. A recent report on *Arundo donax* (Haworth et al., 2018), showed that water stress increases the rapidity of stomatal closure and reduced the speed of opening, consistent with our data in wheat. Similarly, in Lawson and Blatt (2014), *Vicia faba* plants subjected to water stress showed a faster *g_s_* reduction during a shade fleck whilst a slower *g_s_* increase was recorded for a sun fleck. However, recent work by Gerardin et al. (2018) reported an increase in rapidity for both the opening and the closing phase in *Nicotiana tabacum* under reduced water availability. It should be noted that in *N. tabacum* a strong asymmetry between the opening and closing phase (due to a faster closing phase) under control conditions was also reported. Under optimal soil water availability, asymmetric stomatal responses have not been previously described in wheat (e.g., McAusland et al., 2016), thus suggesting that the opening/closing ratio under optimal growth conditions might be species-specific and strongly influenced by water status. Thus, the presence of asymmetric stomatal responses under stress conditions could be considered as: (1) an adaptation to reduce water loss (stronger coordination between *A* and *g_s_*) and (2) a mechanism to limit increasing *g_s_* after steady state *A* has been achieved (McAusland et al., 2016). Our data suggests that both possibilities are conceivable under reduced water availability, with both high *K_i_* and low *K_d_* values observed in wheat. Water stress therefore exacerbates conservative responses under dynamic light in wheat allowing further opportunities for adaptation to reduced water availability conditions.

Only a handful of studies have examined the effect of atmospheric [CO_2_] on stomatal kinetics, with most research focusing on the effects for steady stage *g_s_* or changes in stomatal anatomy (Ainsworth and Rogers, 2007). Leakey et al. (2002) reported that in *Shorea leprosula*, the relative enhancement of biomass driven by elevated [CO_2_] was greater under dynamic irradiance compared to uniform irradiance. Consistent with our findings in wheat, Leakey et al. (2002) suggested that faster stomatal opening under dynamic conditions reduced the time to reach maximum *g_s_* and reduced CO_2_ limitation of *A*. Therefore, a faster stomatal opening phase (in response to an increase in irradiance) might be a leaf trait that has an additional positive effect under elevated [CO_2_] that deserves further investigation at the field level. Further efforts should focus on understanding and quantifying the effects of these major environmental factors on stomatal dynamics under fluctuating light environments.

## Conclusion

To our knowledge, this is the first report showing significant genotypic variation in wheat for the rapidity of stomatal responses. Our work illustrates that slow *g_s_* responses can limit *A* during a low to high light transition by 7–15%, while slow reduction of *g_s_* during a high to low light transition strongly limits water conservation. Measurements obtained post-anthesis suggest that leaf age might exacerbate stomatal limitations by reducing the rapidity of stomatal responses, whilst environmental cues (i.e., water stress and [CO_2_]) also affected this. Evidence of significant genotypic variation for these traits highlights them as novel and as yet unexploited targets for crop improvement programs, which aim to develop cultivars that maximize photosynthesis and minimize the waste of water in the dynamic light environments encountered in the field. This work lends to a greater understanding of the interactions between stomatal behavior, environmental cues and leaf performance, which guides the establishment of ideotypes for specific growth environments. For example, the cultivar Hereward demonstrated fast *g_s_* responses at GS 31 and 41 and minimal limitation of *A*, with potential for exploitation to provide ideotypes for environments in which conservation of water use is a priority. On the other hand, cultivars such as Soissons and Xi19 demonstrated high photosynthetic capacity, high overall *g_s_* values, but slow *g_s_* responses, traits that may be useful for high-light and high-temperature environments. Improvement of stomatal responses under a dynamic light environment might support the optimization of resource use and yield in major crops, and therefore inform the development of new crop ideotypes with higher yield potential and resilience to future environmental changes.

## Author Contributions

MF and TL design the experiments, analyzed the data, and wrote the manuscript. MF executed all the experiments and acquired all the data. SW analyzed stomatal density and pore length. JC, EO, AG, CR, and JVR helped with interpretation of data and edit the manuscript. TL, JC, EO, and CR acquired project funding and resources.

## Conflict of Interest Statement

The authors declare that the research was conducted in the absence of any commercial or financial relationships that could be construed as a potential conflict of interest.

## Footnotes

1 http://www.r-project.org/

## Supplementary Material

TABLE S1 Duncan’s multiple comparisons test output carried out for the gas-exchange data in Experiment 1. The test was performed on both “cultivar” and “stage” factors. In the analysis of the factor “stage”, vegetative, flag, and heading represents GS31, 41, and 71 respectively.
