# Supplementary material for: Genotypic, Developmental and Environmental Effects on the Rapidity of gs in Wheat: Impacts on Carbon Gain and Water-Use Efficiency
Source: Front Plant Sci. 2019 Apr 17;10:492. doi: 10.3389/fpls.2019.00492 (PMC6479173; doi:10.3389/fpls.2019.00492)
Supplement: TABLE S1 — Duncan’s multiple comparisons test output carried out for the gas-exchange data in Experiment 1. The test was performed on both “cultivar” and “stage” factors. In the analysis of the factor “stage”, vegetative, flag, and heading represents GS31, 41, and 71 respectively. [file Table_1.DOCX]

|  |  |  |  |  |  |  |  |  |  |  |  |
| --- | --- | --- | --- | --- | --- | --- | --- | --- | --- | --- | --- |
|  |  |  |  |  |  |  |  |  |  |  |  |
|  |  |  |  |  |  |  |  |  |  |  |  |
| A | Stage |  |  |  |  |  |  |  |  |  |  |
| Duncan a,b,c | |  |  |  |  |  |  |  |  |  |  |
| A | N | Subset |  |  |  |  |  |  |  |  |  |
|  |  | 1 | 2 | 3 | Levels |  |  |  |  |  |  |
| Heading | 29 | 20.56 |  |  | a |  |  |  |  |  |  |
| Vegetative | 39 |  | 22.72 |  | b |  |  |  |  |  |  |
| Flag | 44 |  |  | 29.2 | c |  |  |  |  |  |  |
| Sig. |  | 1 | 1 | 1 |  |  |  |  |  |  |  |
|  |  |  |  |  |  |  |  |  |  |  |  |
|  |  |  |  |  |  |  |  |  |  |  |  |
| A | Cultivar |  |  |  |  |  |  |  |  |  |  |
| Duncan a,b,c | |  |  |  |  |  |  |  |  |  |  |
| A | N | Subset |  |  |  |  |  |  |  |  |  |
|  |  | 1 | 2 | 3 | 4 | 5 | Levels |  |  |  |  |
| Hereward | 15 | 20.48 |  |  |  |  | a |  |  |  |  |
| Rialto | 11 | 21.34 | 21.34 |  |  |  | ab |  |  |  |  |
| Claire | 14 |  | 23.21 | 23.21 |  |  | bc |  |  |  |  |
| Robigus | 15 |  | 23.92 | 23.92 |  |  | bc |  |  |  |  |
| Alchemy | 14 |  |  | 25.49 |  |  | c |  |  |  |  |
| Brompton | 15 |  |  | 25.85 | 25.85 |  | cd |  |  |  |  |
| Xi19 | 14 |  |  |  | 28.28 | 28.28 | de |  |  |  |  |
| Soissons | 14 |  |  |  |  | 28.64 | e |  |  |  |  |
| Sig. |  | 0.493 | 0.052 | 0.055 | 0.055 | 0.776 |  |  |  |  |  |
|  |  |  |  |  |  |  |  |  |  |  |  |
|  |  |  |  |  |  |  |  |  |  |  |  |
| gs | Stage |  |  |  |  |  |  |  |  |  |  |
| Duncan a,b,c | |  |  |  |  |  |  |  |  |  |  |
| gs | N | Subset |  |  |  |  |  |  |  |  |  |
|  |  | 1 | 2 | Levels |  |  |  |  |  |  |  |
| Vegetative | 39 | 0.43 |  | a |  |  |  |  |  |  |  |
| Heading | 29 | 0.45 |  | a |  |  |  |  |  |  |  |
| Flag | 44 |  | 0.57 | b |  |  |  |  |  |  |  |
| Sig. |  | 0.254 | 1 |  |  |  |  |  |  |  |  |
|  |  |  |  |  |  |  |  |  |  |  |  |
|  |  |  |  |  |  |  |  |  |  |  |  |
| gs | Cultivar |  |  |  |  |  |  |  |  |  |  |
| Duncan a,b,c | |  |  |  |  |  |  |  |  |  |  |
| gs | N | Subset |  |  |  |  |  |  |  |  |  |
|  |  | 1 | 2 | 3 | 4 | Levels |  |  |  |  |  |
| Rialto | 11 | 0.39 |  |  |  | a |  |  |  |  |  |
| Hereward | 15 | 0.43 | 0.43 |  |  | ab |  |  |  |  |  |
| Claire | 14 |  | 0.47 | 0.47 |  | bc |  |  |  |  |  |
| Robigus | 15 |  | 0.48 | 0.48 |  | bc |  |  |  |  |  |
| Alchemy | 14 |  | 0.49 | 0.49 |  | bc |  |  |  |  |  |
| Brompton | 15 |  | 0.5 | 0.5 |  | bc |  |  |  |  |  |
| Xi19 | 14 |  |  | 0.53 | 0.53 | cd |  |  |  |  |  |
| Soissons | 14 |  |  |  | 0.58 | d |  |  |  |  |  |
| Sig. |  | 0.208 | 0.07 | 0.075 | 0.122 |  |  |  |  |  |  |
|  |  |  |  |  |  |  |  |  |  |  |  |
|  |  |  |  |  |  |  |  |  |  |  |  |
|  |  |  |  |  |  |  |  |  |  |  |  |
| Ki | Stage |  |  |  |  |  |  |  |  |  |  |
| Duncan a,b,c | |  |  |  |  |  |  |  |  |  |  |
| Ki | N | Subset |  |  |  |  |  |  |  |  |  |
|  |  | 1 | 2 | Levels |  |  |  |  |  |  |  |
| Flag | 44 | 11.5 |  | a |  |  |  |  |  |  |  |
| Vegetative | 39 | 11.76 |  | a |  |  |  |  |  |  |  |
| Heading | 29 |  | 18.49 | b |  |  |  |  |  |  |  |
| Sig. |  | 0.834 | 1 |  |  |  |  |  |  |  |  |
|  |  |  |  |  |  |  |  |  |  |  |  |
|  |  |  |  |  |  |  |  |  |  |  |  |
| Ki | Cultivar |  |  |  |  |  |  |  |  |  |  |
| Duncan a,b,c | |  |  |  |  |  |  |  |  |  |  |
| KI | N | Subset |  |  |  |  |  |  |  |  |  |
|  |  | 1 | 2 | Levels |  |  |  |  |  |  |  |
| Robigus | 15 | 10.35 |  | a |  |  |  |  |  |  |  |
| Claire | 14 | 12.4 | 12.4 | ab |  |  |  |  |  |  |  |
| Xi19 | 14 | 12.57 | 12.57 | ab |  |  |  |  |  |  |  |
| Hereward | 15 | 12.88 | 12.88 | ab |  |  |  |  |  |  |  |
| Soissons | 14 | 13.47 | 13.47 | ab |  |  |  |  |  |  |  |
| Rialto | 11 |  | 15.12 | b |  |  |  |  |  |  |  |
| Brompton | 15 |  | 15.19 | b |  |  |  |  |  |  |  |
| Alchemy | 14 |  | 15.71 | b |  |  |  |  |  |  |  |
| Sig. |  | 0.166 | 0.154 |  |  |  |  |  |  |  |  |
|  |  |  |  |  |  |  |  |  |  |  |  |
|  |  |  |  |  |  |  |  |  |  |  |  |
| Kd Stage |  |  |  |  |  |  |  |  |  |  |  |
| Duncan a,b,c | |  |  |  |  |  |  |  |  |  |  |
| Kd | N | Subset |  |  |  |  |  |  |  |  |  |
|  |  | 1 | 2 | Levels |  |  |  |  |  |  |  |
| Flag | 44 | 9.44 |  | a |  |  |  |  |  |  |  |
| Vegetative | 39 | 11.59 |  | a |  |  |  |  |  |  |  |
| Heading | 29 |  | 15.4 | b |  |  |  |  |  |  |  |
| Sig. |  | 0.12 | 1 |  |  |  |  |  |  |  |  |
|  |  |  |  |  |  |  |  |  |  |  |  |
|  |  |  |  |  |  |  |  |  |  |  |  |
| Kd | Cultivar |  |  |  |  |  |  |  |  |  |  |
| Duncan a,b,c | |  |  |  |  |  |  |  |  |  |  |
| Kd | N | Subset |  |  |  |  |  |  |  |  |  |
|  |  | 1 |  |  |  |  |  |  |  |  |  |
| Claire | 14 | 10.08 |  |  |  |  |  |  |  |  |  |
| Robigus | 15 | 10.13 |  |  |  |  |  |  |  |  |  |
| Brompton | 15 | 10.99 |  |  |  |  |  |  |  |  |  |
| Rialto | 11 | 11.01 |  |  |  |  |  |  |  |  |  |
| Xi19 | 14 | 11.3 |  |  |  |  |  |  |  |  |  |
| Hereward | 15 | 12.68 |  |  |  |  |  |  |  |  |  |
| Soissons | 14 | 13.73 |  |  |  |  |  |  |  |  |  |
| Alchemy | 14 | 13.9 |  |  |  |  |  |  |  |  |  |
| Sig. |  | 0.146 |  |  |  |  |  |  |  |  |  |
|  |  |  |  |  |  |  |  |  |  |  |  |
|  |  |  |  |  |  |  |  |  |  |  |  |
|  |  |  |  |  |  |  |  |  |  |  |  |
| Time to reach A | | Stage |  |  |  |  |  |  |  |  |  |
| Duncan a,b,c | |  |  |  |  |  |  |  |  |  |  |
| Time to reach A | N | Subset |  |  |  |  |  |  |  |  |  |
|  |  | 1 | 2 | 3 | Levels |  |  |  |  |  |  |
| Vegetative | 39 | 9.6 |  |  | a |  |  |  |  |  |  |
| Flag | 44 |  | 12.45 |  | b |  |  |  |  |  |  |
| Heading | 29 |  |  | 18.3 | c |  |  |  |  |  |  |
| Sig. |  | 1 | 1 | 1 |  |  |  |  |  |  |  |
|  |  |  |  |  |  |  |  |  |  |  |  |
|  |  |  |  |  |  |  |  |  |  |  |  |
| Time to reach A | | Cultivar |  |  |  |  |  |  |  |  |  |
| Duncan a,b,c | |  |  |  |  |  |  |  |  |  |  |
| Time to reach A | N | Subset |  |  |  |  |  |  |  |  |  |
|  |  | 1 | 2 | Levels |  |  |  |  |  |  |  |
| Claire | 14 | 10.89 |  | a |  |  |  |  |  |  |  |
| Hereward | 15 | 11.04 |  | a |  |  |  |  |  |  |  |
| Robigus | 15 | 12.48 | 12.48 | ab |  |  |  |  |  |  |  |
| Soissons | 14 | 13.17 | 13.17 | ab |  |  |  |  |  |  |  |
| Xi19 | 14 | 13.41 | 13.41 | ab |  |  |  |  |  |  |  |
| Brompton | 15 | 13.71 | 13.71 | ab |  |  |  |  |  |  |  |
| Rialto | 11 | 13.74 | 13.74 | ab |  |  |  |  |  |  |  |
| Alchemy | 14 |  | 15.63 | b |  |  |  |  |  |  |  |
| Sig. |  | 0.181 | 0.13 |  |  |  |  |  |  |  |  |
|  |  |  |  |  |  |  |  |  |  |  |  |
|  |  |  |  |  |  |  |  |  |  |  |  |
| Limitation of A by gs |  |  |  |  |  |  |  |  |  |  |  |
| Duncan a,b,c stage | |  |  |  |  |  |  |  |  |  |  |
| Limitation of A by gs | N | Subset |  |  |  |  |  |  |  |  |  |
|  |  | 1 | 2 | Levels |  |  |  |  |  |  |  |
| GS 31 | 48 | 7.7337 |  | a |  |  |  |  |  |  |  |
| GS 45 | 48 |  | 9.84 | b |  |  |  |  |  |  |  |
| GS 71 | 30 |  | 10.8889 | b |  |  |  |  |  |  |  |
| Sig. |  | 1 | 0.273 |  |  |  |  |  |  |  |  |
|  |  |  |  |  |  |  |  |  |  |  |  |
|  |  |  |  |  |  |  |  |  |  |  |  |
| Limitation of A by gs | | Cultivar |  |  |  |  |  |  |  |  |  |
| Duncan a,b,c | |  |  |  |  |  |  |  |  |  |  |
| Limitation of A by gs | N | Subset |  |  |  |  |  |  |  |  |  |
|  |  | 1 | 2 | Levels |  |  |  |  |  |  |  |
| Hereward | 16 | 8.0448 |  | a |  |  |  |  |  |  |  |
| Claire | 16 | 8.2031 | 8.2031 | ab |  |  |  |  |  |  |  |
| Robigus | 16 | 8.9078 | 8.9078 | ab |  |  |  |  |  |  |  |
| Rialto | 16 | 8.92 | 8.92 | ab |  |  |  |  |  |  |  |
| Brompton | 16 | 9.3483 | 9.3483 | ab |  |  |  |  |  |  |  |
| Xi19 | 15 | 9.5552 | 9.5552 | ab |  |  |  |  |  |  |  |
| Soissons | 15 | 9.933 | 9.933 | ab |  |  |  |  |  |  |  |
| Alchemy | 16 |  | 11.4435 | b |  |  |  |  |  |  |  |
| Sig. |  | 0.655 | 0.064 |  |  |  |  |  |  |  |  |
|  |  |  |  |  |  |  |  |  |  |  |  |
|  |  |  |  |  |  |  |  |  |  |  |  |
| Time to restore WUE | Stage |  |  |  |  |  |  |  |  |  |  |
| Duncan a,b,c | |  |  |  |  |  |  |  |  |  |  |
| Time to restore WUE | N | Subset |  |  |  |  |  |  |  |  |  |
|  |  | 1 | 2 | Levels |  |  |  |  |  |  |  |
| GS31 | 47 | 22.89 |  | a |  |  |  |  |  |  |  |
| GS41 | 48 |  | 31.21 | b |  |  |  |  |  |  |  |
| GS71 | 29 |  | 31.82 | b |  |  |  |  |  |  |  |
| Sig. |  | 1 | 0.901 |  |  |  |  |  |  |  |  |
|  |  |  |  |  |  |  |  |  |  |  |  |
|  |  |  |  |  |  |  |  |  |  |  |  |
| Time to restore WUE | | Cultivar |  |  |  |  |  |  |  |  |  |
| Duncan a,b,c | |  |  |  |  |  |  |  |  |  |  |
| Time to restore WUE | N | Subset |  |  |  |  |  |  |  |  |  |
|  |  | 1 | 2 | Levels |  |  |  |  |  |  |  |
| Hereward | 16 | 25.78 |  | a |  |  |  |  |  |  |  |
| Brompton | 16 | 26.19 | 26.19 | ab |  |  |  |  |  |  |  |
| Claire | 16 | 26.19 | 26.19 | ab |  |  |  |  |  |  |  |
| Robigus | 16 | 27.03 | 27.03 | ab |  |  |  |  |  |  |  |
| Xi19 | 15 | 27.85 | 27.85 | ab |  |  |  |  |  |  |  |
| Rialto | 16 | 28.75 | 28.75 | ab |  |  |  |  |  |  |  |
| Alchemy | 14 | 31.48 | 31.48 | ab |  |  |  |  |  |  |  |
| Soissons | 15 |  | 33.02 | b |  |  |  |  |  |  |  |
| Sig. |  | 0.188 | 0.056 |  |  |  |  |  |  |  |  |
|  |  |  |  |  |  |  |  |  |  |  |  |
|  |  |  |  |  |  |  |  |  |  |  |  |
| limitation of WUE by gs | Stage |  |  |  |  |  |  |  |  |  |  |
| Duncan a,b,c | |  |  |  |  |  |  |  |  |  |  |
| limitation of WUE by gs | N | Subset |  |  |  |  |  |  |  |  |  |
|  |  | 1 | 2 | Levels |  |  |  |  |  |  |  |
| GS31 | 47 | 47.42 |  | a |  |  |  |  |  |  |  |
| GS41 | 48 |  | 60.52 | b |  |  |  |  |  |  |  |
| GS71 | 29 |  | 63.53 | b |  |  |  |  |  |  |  |
| Sig. |  | 1 | 0.442 |  |  |  |  |  |  |  |  |
|  |  |  |  |  |  |  |  |  |  |  |  |
|  |  |  |  |  |  |  |  |  |  |  |  |
| limitation of WUE by gs | Cultivar |  |  |  |  |  |  |  |  |  |  |
| Duncan a,b,c | |  |  |  |  |  |  |  |  |  |  |
| limitation of WUE by gs | N | Subset |  |  |  |  |  |  |  |  |  |
|  |  | 1 | 2 | Levels |  |  |  |  |  |  |  |
| Rialto | 16 | 49.42 |  | a |  |  |  |  |  |  |  |
| Brompton | 16 | 52.84 | 52.84 | ab |  |  |  |  |  |  |  |
| Hereward | 16 | 54.29 | 54.29 | ab |  |  |  |  |  |  |  |
| Robigus | 16 | 54.77 | 54.77 | ab |  |  |  |  |  |  |  |
| Xi19 | 15 | 56.41 | 56.41 | ab |  |  |  |  |  |  |  |
| Soissons | 15 | 59.25 | 59.25 | ab |  |  |  |  |  |  |  |
| Claire | 16 | 61.36 | 61.36 | ab |  |  |  |  |  |  |  |
| Alchemy | 14 |  | 62.73 | b |  |  |  |  |  |  |  |
| Sig. |  | 0.057 | 0.197 |  |  |  |  |  |  |  |  |
