# Supplementary material for: Genotypic, Developmental and Environmental Effects on the Rapidity of gs in Wheat: Impacts on Carbon Gain and Water-Use Efficiency
Source: Front Plant Sci. 2019 Apr 17;10:492. doi: 10.3389/fpls.2019.00492 (PMC6479173; doi:10.3389/fpls.2019.00492)
Supplement: Supplementary file 2 [file Table_2.DOCX]

**Supplementary**

**
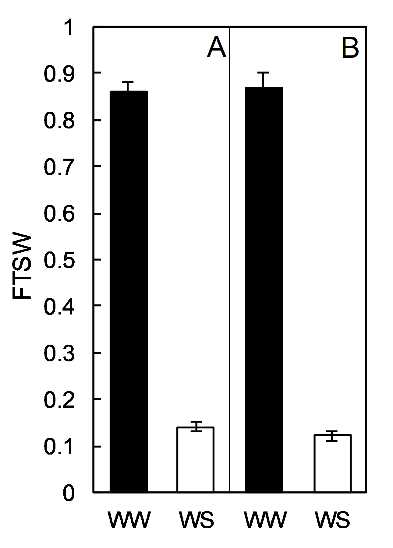
**

Supplementary 1. Fraction of transpirable soil water (FTSW) for well-watered (WW) and water-stressed (WS) wheat plants (cv. Soissons). A and B represent the FTSW values at the time of the step-change analysis for the two different cycles of water stress. Error bars represent ± SEM (n=3).


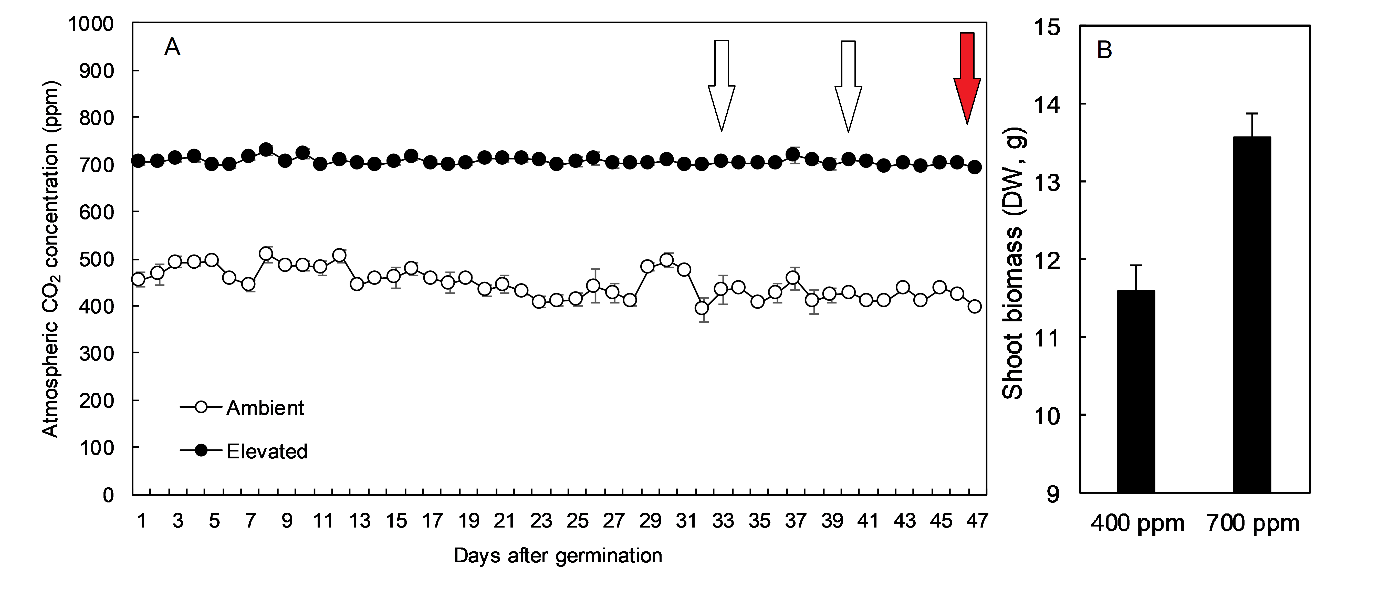


Supplementary 2. A) Atmospheric CO_2_ concentration for ambient and elevated experimental conditions. Error bars represent ± SEM (n=3). Data were collected throughout the experiment until harvest. White harrows represent the time frame used for gas-exchange analysis, red harrow represent shoot harvest. B) Shoot biomass of wheat plants (cv. Soissons) subjected to ambient and elevated [CO_2_] for 48 days from sowing. Error bars represent ± SEM (n=6).
